# A Cohort Study of Adult Patients with Severe Dengue in Taiwanese Intensive Care Units: The Elderly and APTT Prolongation Matter for Prognosis

**DOI:** 10.1371/journal.pntd.0005270

**Published:** 2017-01-06

**Authors:** Chih-Cheng Hsieh, Cong-Tat Cia, Jen-Chieh Lee, Junne-Ming Sung, Nan-Yao Lee, Po-Lin Chen, Te-Hui Kuo, Jo-Yen Chao, Wen-Chien Ko

**Affiliations:** 1 Division of Critical Care Medicine, Department of Internal Medicine, National Cheng Kung University Hospital, College of Medicine, National Cheng Kung University, Tainan, Taiwan; 2 Division of Nephrology, Department of Internal Medicine, National Cheng Kung University Hospital, College of Medicine, National Cheng Kung University, Tainan, Taiwan; 3 Division of Infectious Diseases, Department of Internal Medicine, National Cheng Kung University Hospital, College of Medicine, National Cheng Kung University, Tainan, Taiwan; 4 Department and Graduate Institute of Public Health, College of Medicine, National Cheng Kung University, Tainan, Taiwan; Centre Hospitalier Universitaire de la Réunion, RÉUNION

## Abstract

**Background:**

There was a large dengue outbreak in Taiwan in 2015, in which the ages of the affected individuals were higher than those in other countries. The aim of this study was to explore the characteristics and prognostic factors for adults with severe dengue in intensive care units (ICUs).

**Methods:**

All adults admitted to ICUs with dengue virus infection (DENV) at a medical center from July 1, 2015 to December 31, 2015 were enrolled. DENV was diagnosed by the presence of serum NS1 antigen, IgM antibodies to dengue virus, or dengue virus RNA by real-time reverse transcriptase polymerase chain reaction. Demographic data, clinical features, and lab data were collected, and a multivariate Cox model was used to identify the predictive factors for in-hospital mortality.

**Results:**

Seventy-five patients admitted to ICUs with laboratory-confirmed DENV were enrolled (mean age 72.3±9.3 years). The most common comorbidities included hypertension (72.0%), diabetes (43.7%), and chronic kidney disease (22.7%). The in-hospital case fatality rate (CFR) was 41.3%. The patients who died were predominantly female, had higher disease severity at ICU admission, shorter ICU/hospital stay, longer initial activated partial thromboplastin time (APTT), and higher initial serum aspartate transaminase levels. Cardiac arrest before ICU admission (hazard ratio [HR]: 6.26 [1.91–20.54]), prolonged APTT (>48 seconds; HR: 3.91 [1.69–9.07]), and the presence of acute kidney injury on admission (HR: 2.48 [1.07–5.74]), were independently associated with in-hospital fatality in the Cox multivariate analysis.

**Conclusion:**

During the 2015 dengue outbreak in Taiwan, the patients with severe dengue in ICUs were characterized by old age, multiple comorbidities, and a high CFR. Organ failure (including cardiac failure, and renal failure) and coagulation disturbance (prolongation of initial APTT) were independent predictive factors for in-hospital fatality.

## Author Summary

Severe forms of dengue fever (DF) are usually considered as a pediatric disease in southern Asia and are graded from dengue hemorrhagic fever (DHF) to dengue with shock syndrome (DSS). However, the age of affected individuals is increasing in Singapore, Thailand, Mainland China, Taiwan and many other countries. Limited data are available on the elderly with DF in intensive care units (ICUs). DF in the elderly is an emerging infectious disease and poses a new clinical challenge to physicians. We enrolled the patients with laboratory-confirmed dengue in our ICU during the 2015 dengue outbreak in Taiwan. These patients were characterized by old age, multiple comorbidities, and a high case fatality rate (CFR). All of the patients were classified as severe dengue, and the in-hospital CFR was 41.3%. Renal failure and cardiac arrest were associated with fatality. In addition to organ failure, initial prolonged activated partial thromboplastin time (APTT) in our study was consistent with an independent predictive factor for in-hospital fatality. Previous studies have also reported that prolongation of APTT is a clinical predictor of dengue virus infection (DENV) or DHF. Our results highlight that APTT prolongation may be a prognostic factor in critically ill adults with severe dengue.

## Introduction

Dengue fever (DF) is a mosquito-borne disease that affects at least 50 million people annually worldwide [1, 2]. There have been several outbreaks during the past three decades in Taiwan [3], most recently, in southern Taiwan in 2015 which involved 43,784 cases [4]. DF is often considered to be a pediatric disease in Southeast Asia [5]. However, severe dengue can also occur in adults and the elderly [6–8]. The age of DF cases in Taiwan is generally older than that in other countries [9–13]. The predominant age of patients with DF in the Philippines during 2000 to 2009 was 5–14 years [11], and 24 years in Thailand during 2001 to 2011 [13]. In comparison, the median age of Taiwanese patients with DF was 45 years from 2010 to 2012 with the major age group being older than 65 years [12]. Old patients with DF are often associated with atypical presentations, longer hospitalization and more underlying diseases [14]. In particular, diabetes mellitus, hypertension, and aging are associated with a higher risk of DHF/DSS and a worse outcome [6, 15–18].

Various predictive factors for mortality have been identified in DF, including old age, acute renal injury, acute respiratory failure, hepatitis, altered mental status, bleeding and coagulopathy [19–21]. The acute physiology and chronic health evaluation (APACHE) II score may predict mortality in patients admitted to an intensive care unit (ICU) in a prospective observational cohort study [22]. Recently a Brazilian study also showed that APACHE II score and sequential organ failure assessment (SOFA) score were associated with in-hospital fatality in patients in an ICU [23]. During the dengue outbreak in 2015, many elderly patients were admitted to ICUs. Therefore, the aim of this study was to explore the clinical characteristics and prognostic factors among this elderly cohort in our ICUs in Taiwan.

## Methods

### Case identification

There are eight ICUs with a total of 104 beds at National Cheng Kung University Hospital, a tertiary hospital in southern Taiwan. The medical records of all patients with DF admitted to our ICUs from July 1, 2015 and December 31, 2015 were reviewed. The diagnosis of DENV was confirmed by one or more of the following tests: presence of serum dengue NS1 antigen, dengue IgM antibodies as detected using a Bioline Dengue Duo^™^ kit (Standard Diagnostics, Korea), or dengue virus RNA detected by real-time reverse transcriptase-polymerase chain reaction (RT-PCR) (TIB Molbiol, Lightmix kit; Roche Applied Science, Berlin, Germany).

### Data collection

Demographic data, underlying diseases, admission characteristics, and laboratory data were collected through the ICU Clinical Information System (IntelliVue Clinical Information Portfolio, Philips) and electronic medical records. The dengue severity was evaluated at hospital admission and ICU admission according to the dengue classification of the World Health Organization (WHO) 2009 [24]. Acute kidney injury (AKI) at ICU admission was defined according to the Kidney Disease: Improve Global Outcomes (KDIGO) clinical practice guidelines for AKI in the first 24 hours after ICU admission [25]. SOFA and APACHE II scores were calculated in the first 24 hours after ICU admission [26, 27]. Bacteremia and fungemia were defined by the presence of clinically significant microorganism during the hospital course. Laboratory data were recorded on the first day of admission to the ICU. All data were delinked, and are provided in the S1 Dataset.

### Ethical concerns

This study adhered to the Declaration of Helsinki and was approved by the Human Research and Ethics Committee of National Cheng Kung University Hospital (IRB number: A-ER-104-367). The Institutional Review Board waived the need for informed consent.

### Statistical analysis

Continuous variables were tested for normal distribution using the Shapiro-Wilk test. Normally distributed variables were reported as means with standard deviation (SD) and compared using the Student’s *t*-test. Non-normally distributed continuous variables were reported as medians with inter-quartile range (IQR) and compared using the Mann-Whitney U-test. Categorical data were reported as proportions and compared using the Fisher’s exact test. A univariate Cox model was used to identify the possible predictive factors for death. Receiver operating characteristic (ROC) curves were constructed for the severity scores and the continuous variables of significant prognostic factors. The cut-off value for each significant continuous variable was selected using the Youden index. The cases with missing data were excluded in the Cox model analysis. A multivariate Cox model with binary variables was used to identify the predictive factors for death. Most data were analyzed using SPSS Statistical software for Windows, version 17.0 (SPSS Inc., Chicago). The comparison of ROC curves was analyzed using MedCalc, version 12.7.8.0 with DeLong test [28]. A two-sided *P* value of less than 0.05 was considered to be statistically significant.

## Results

**Fig 1:**
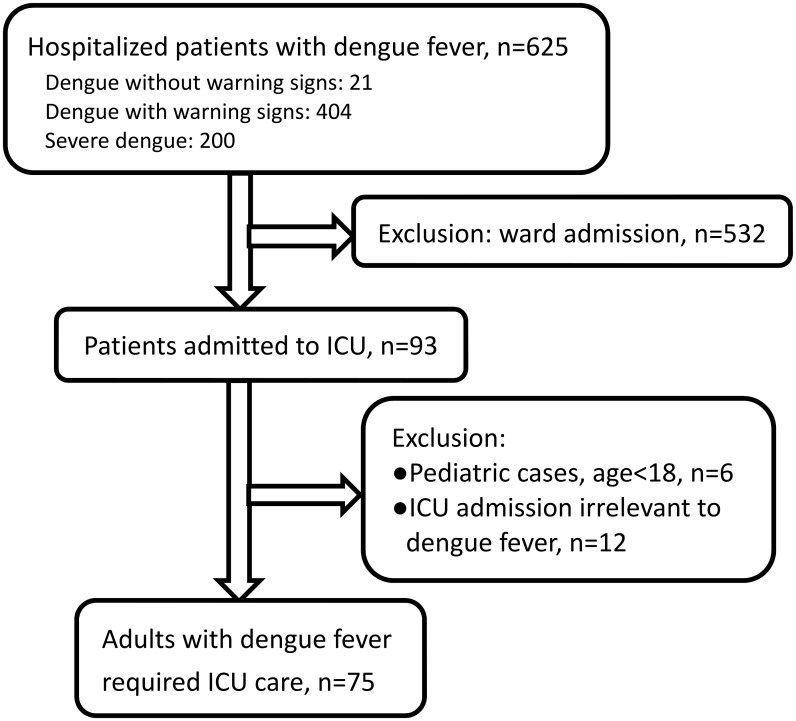
Patient inclusion flowchart.

**Fig 2:**
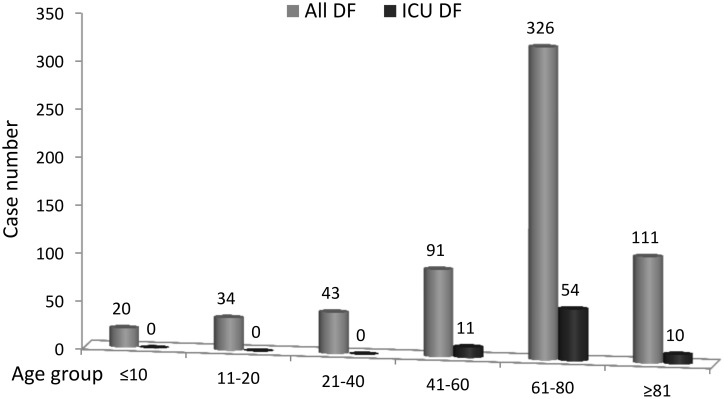
The age distribution of hospitalized patient with dengue fever and of those admitted to ICUs.

Six hundred and twenty-five inpatients with laboratory-confirmed dengue were identified during the 6-month study period. Of these patients, 532 patients were excluded because they were admitted to a general ward only, and 93(14.9%) patients were admitted to an ICU during the study period. Among them, six patients were younger than 18 years old, and the ICU course was irrelevant to dengue infection in 12. Finally, 75 adult patients with laboratory-confirmed DENV were included (Fig 1). Age distribution of all hospitalized patients with dengue fever and of those admitted to ICUs was shown in Fig 2. Sixty-two (82.7%) patients had NS1 antigen detected in their serum, 29 (38.6%) had a positive PCR for dengue virus RNA, and 25 (33.3%) had IgM antibodies to dengue virus. Thirty-three (44.0%) patients had at least two positive results of these three tests. The overall age distribution of dengue in Taiwan during the previous year and this outbreak is listed in S1 Fig.

Fourteen (18.7%) patients were classified as having dengue with warning signs, and the other 61 (81.3%) patients were classified as having severe dengue at hospital admission. All patients were classified as having severe dengue at ICU admission according to the dengue classification of World Health Organization (WHO) 2009 [24]. The primary indications for ICU admission included severe gastrointestinal bleeding (23 cases, 30.7%), acute respiratory failure (18, 24.0%), and shock (10, 13.3%). Other indications for ICU admission included encephalopathy (eight, 10.7%), post cardiac arrest (five, 6.7%), intracranial hemorrhage (five, 6.7%), myocarditis (four, 5.3%), and severe hepatitis (two, 2.6%).

The mean age of the 75 patients was 72.3±9.3 years (ranging from 50.7 to 88.5 years, Table 1). Males accounted for 61.3% of the patients. The most common comorbidities included hypertension (72.0%), diabetes (43.7%), and chronic kidney disease (22.7%). Median APACHE II score and SOFA score at the ICU admission was 20 and 11, respectively. At the ICU admission, 41 (54.7%) patients had AKI, according to the KDIGO criteria and 12 (29.3%) of whom needed renal replacement therapy during the ICU stay. Four (5.3%) patients had end-stage renal disease and received maintenance dialysis. Approximately three-quarters (57, 76.0%) of all patients required ventilator support during the ICU stay. Seven (9.3%) patients experienced cardiac arrest before ICU admission. The median resuscitation time was 10 minutes, ranging from 2–38 minutes. Five of them were admitted to the ICU immediately after the resuscitation. One experienced cardiac arrest during the endotracheal intubation at the second hour of emergency department (ED) arrival and was admitted to the ICU on the next day. The another one experienced cardiac arrest due to massive gastrointestinal bleeding and severe metabolic acidosis at the 23^rd^ hour of ED arrival and was admitted to the ICU at the 45^th^ hour of ED arrival. Fifty-four (72.9%) patients received blood transfusions (Table 1).

**Table 1:** Demographics and clinical characteristics of the 75 adults with severe dengue in intensive care units.

Variables	All (n = 75)	Survivors (n = 44)	Non-survivors (n = 31)	*P* value
**Age (years)**	72.3±9.3	70.8±8.9	74.6±9.4	0.070
**Male**	46 (61.3)	32 (72.7)	14 (45.2)	0.029
**Body weight (kg)**	64.9±12.4	67.3±10.7	61.6±14.0	0.064
**Underlying disease**				
** Hypertension**	54 (72.0)	35 (79.5)	19 (61.3)	0.117
** Diabetes mellitus**	32 (42.7)	18 (40.9)	14 (45.2)	0.814
** Chronic kidney disease**	17 (22.7)	8 (18.2)	9 (29.0)	0.281
** Dyslipidemia**	16 (21.3)	9 (20.5)	7 (22.6)	1
** Coronary artery disease**	14 (18.7)	8 (18.2)	6 (19.4)	1
** Malignancy**	11 (14.7)	5 (11.4)	6 (19.4)	0.509
** Stroke**	10 (13.3)	7 (15.9)	3 (9.7)	0.509
** Chronic lung disease**	7 (9.3)	6 (13.6)	1 (3.2)	0.228
** Heart failure**	7 (9.3)	4 (9.1)	3 (9.7)	1
** Liver disease**	5 (6.7)	3 (6.8)	2 (6.5)	1
**Severe dengue at presentation**	61(81.3)	32(72.7)	29(93.5)	0.034
**ICU parameters**				
** APACHE II score**	20 [16,28]	17 [13,22]	27 [21,42]	<0.001
** SOFA score**	11 [7,15]	8 [5,12.8]	16 [11,19]	<0.001
**Cardiac arrest before ICU admission**	7 (9.3)	1 (2.3)	6 (19.4)	0.018
**Acute kidney injury at ICU admission**	41 (54.7)	18 (40.9)	23 (74.2)	0.005
**Renal replacement therapy**	12 (16.9)	4 (9.1)	8 (25.8)	0.063
**Respiratory failure at ICU admission**	47(62.7)	22(50)	25(80.6)	0.008
**Mechanical ventilation**	57 (76.0)	28 (63.6)	29 (93.5)	0.003
**Bloodstream infection**	18 (24.0)	8 (18.2)	10 (32.3)	0.180
**Blood transfusion**	54 (72.9)	30 (68.2)	24 (77.4)	0.441
** Platelets**	38 (50.7)	20 (45.5)	18 (58.1)	0.351
** Whole blood**	28 (37.3)	14 (31.8)	14 (45.2)	0.333
** Packed red blood cells**	37 (49.3)	19 (43.2)	18 (58.1)	0.245
** Plasma**	30 (40.0)	14 (31.8)	16 (51.6)	0.099
**Hospital course**				
** ICU stay (days)**	6 [3,14]	9 [5.3,15.8]	4 [1,8]	<0.001
** Hospital stay (days)**	13 [6,23]	20 [11,35.5]	4 [1,8]	<0.001

*Data are expressed as case number (%), mean ± standard deviation, or median [inter-quartile range].

APACHE II = acute physiology and chronic health evaluation II; SOFA = sequential organ failure assessment.

The in-hospital CFR was 41.3% (31 patients), and these patients had a median ICU stay of 4 (IQR: 1–8) days. The leading cause of fatality was multiple organ failure (14/31, 45.2%), and nine of these 14 patients had overt bleeding. Seven (22.6%) patients died of refractory shock and severe metabolic acidosis, six (19.4%) intracranial hemorrhage, three (9.6%) bloodstream infections, and one (3.2%) pneumonia with respiratory failure.

Nineteen bacterial and three fungal pathogens were identified in the bloodstream of 18 (23.7%) patients. Seven patients experienced bloodstream infections early within 48 hours after ICU admission. The etiological species were complex and mainly *Enterobacteriaceae* (40.1%, 9/22). Two patients had polymicrobial bacteremia. Notably, two patients experienced recurrent fever at the eighth and ninth day after the onset of DF, respectively, which was caused by candidemia. Bloodstream infections occurring late in the ICU course were due to the common healthcare-associated pathogens, *Acinetobacter baumannii* (three isolates), *Stenotrophomonas maltophilia* (one), *Chryseobacterium meningosepticum* (one), and *Candida albicans* (one).

Compared with the survivors, the non-survivors were more often females and had a shorter ICU and hospital stay. The rates of underlying diseases and conditions necessitating blood component transfusions were similar between the survivors and non-survivors. The non-survivors had higher APACHE II and SOFA scores and a higher rate of organ failure, including acute respiratory failure and AKI. Thrombocytopenia and elevated levels of serum transaminases were common findings in both groups. The non-survivors had higher initial activated partial thromboplastin time (APTT) than the survivors (50.8 *vs*. 40.8 s, *P* < 0.001), and higher serum levels of aspartate aminotransferase (662.0 *vs*. 240.5 U/L, *P* = 0.042) (Table 2).

**Table 2:** Initial laboratory data of the 75 adults with severe dengue at admission to intensive care units.

Variables	All cases		Survivors		Non-survivors		*P* value
	n	data	n	data	n	data	
**PT (seconds)**	72	13.1 [11.9,15.9]	43	13.1 [11.5,14.3]	29	13.8 [12.0,18.8]	0.241
**APTT (seconds)**	71	44.9±11.1	42	40.8±9.3	29	50.8±10.9	<0.001
**Hemoglobin (g/dL)**	75	12.0±2.8	44	12.4±2.8	31	11.5±2.7	0.194
**Hematocrit (%)**	75	36.7±8.5	44	37.7±8.3	31	35.2±8.7	0.204
**Platelet (x1000/dL)**	75	40.0[15,101]	44	54.5[17,107]	31	23.0[14,59]	0.069
**Total bilirubin (mg/dL)**	60	1.1 [0.8,1.7]	36	1.0 [0.7,1.7]	24	1.2 [0.83,1.78]	0.311
**Creatinine (mg/dL)**	72	1.50 [0.88,2.87]	42	1.02 [0.81,2.65]	30	1.90 [1.00,3.67]	0.057
**AST (U/L)**	71	317.0 [112,1441]	40	230.0 [75.5,1208.0]	31	662.0 [228.0,1642.0]	0.042
**ALT (U/L)**	74	128.0 [47.3,590.3]	43	90.5 [37.3,459.3]	31	297.0 [59.0,622.0]	0.115

Data are expressed as mean ± standard deviation or median [inter-quartile range].

PT = prothrombin time; APTT = activated prothrombin time; AST = aspartate transaminase; ALT = alanine transaminase.

In the univariate Cox model, female gender, cardiac arrest before ICU admission, respiratory failure at ICU admission, high creatinine level, AKI at ICU admission, a high APACHE II or SOFA score, and APTT longer than 48 s were significantly associated with in-hospital fatality. In the multivariate analysis, cardiac arrest before ICU admission (hazard ratio [HR] 6.26 [95% confidence interval: 1.91–20.54]), AKI at ICU admission (HR 2.48 [1.07–5.74]), and initial APTT longer than 48 s (HR 3.91 [1.69–9.07]) were independent predictive factors for in-hospital fatality after adjusting for gender, cardiac arrest before ICU admission, respiratory failure at ICU admission, presence of AKI at ICU admission and initial APTT longer than 48 s (Table 3). APACHE II and SOFA scores include many clinical parameters and were not included in the final model of multivariate Cox analysis as they demonstrated collinearity with other variables. They were positively associated with acute respiratory failure, AKI, and cardiac arrest. Creatinine was also not included in the final model due to collinearity with the presence of AKI at ICU admission. More detailed information about the Cox model construction is included in the S1 File.

**Table 3:** Cox regression analysis of prognostic factors of in-hospital fatality among the 75 adults with severe dengue in intensive care units.

Variables	Unadjusted HR (95% CI)	*P* value	Adjusted HR (95% CI)	*P* value
**Age >70 years**	1.71 (0.74–3.98)	0.211	-	-
**Male**	0.41 (0.20–0.84)	0.015	0.62 (0.30–1.45)	0.298
**APACHE II score >24 points** ^a^	4.70 (2.20–10.03)	<0.001	-	-
**SOFA score >15 points** ^a^	7.56 (3.65–15.64)	<0.001	-	-
**Cardiac arrest before ICU admission**	8.08 (3.06–21.33)	<0.001	6.26 (1.91–20.54)	0.003
**Respiratory failure at ICU admission**	2.73 (1.12–6.67)	0.027	2.69 (0.91–7.95)	0.073
**Acute kidney injury at ICU admission**	2.95 (1.32–6.63)	0.009	2.48 (1.07–5.74)	0.035
**Creatinine >1.5 mg/dL** ^a^	2.52 (1.18–5.39)	0.017	-	-
**APTT >48 seconds** ^b^	4.54 (2.04–10.07)	<0.001	3.91 (1.69–9.07)	0.001

^a^ Variables were not included in the final Cox model due to collinearity.

^b^ Four cases with missing laboratory data were excluded in the final Cox model.

HR = hazard ratio; CI = confidence interval; APTT = activated prothrombin time; APACHE II = acute physiology and chronic health evaluation II; SOFA = sequential organ failure assessment.

Moreover, APTT prolongation > 48 s remained to be an independent predictive factor for in-hospital fatality after adjusting for APACHE II score (HR: 3.34, *P* = 0.006), and was marginally significant after adjusting for SOFA score (HR: 2.43, *P* = 0.053). Detailed information of the Cox regression analysis is listed in the S1 File.

**Fig 3:**
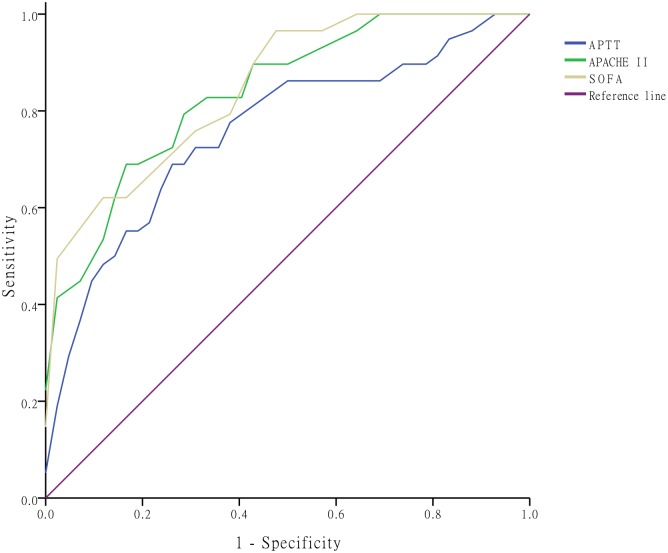
Receiver operating characteristic curves. Receiver operating characteristic curves of the three continuous variables: activated partial thromboplastin time (APTT), acute physiology and chronic health evaluation II (APACHE II), and sequential organ failure assessment (SOFA) scores in predicting in-hospital fatality.

Fig 3 shows the ROC curve with the three continuous variables: APTT, APACHE II score and SOFA score. The area under the ROC curve (AUC) of APTT was 0.76 (95% confidence interval [CI]: 0.64–0.88), APACHE II score 0.84 (0.75–0.93), and SOFA score 0.85 (0.77–0.94). Though there was no significant difference between APTT and two scores (APTT *vs*. APACHE II, *P* = 0.27; APTT *vs*. SOFA, *P* = 0.16), AUCs of APACHE II and SOFA scores, which both were a constellation of multiple clinical and laboratory variables, were higher than that of APTT. However, APTT, a single laboratory parameter, exhibited valuable prognostic significance.

## Discussion

To the best of our knowledge, this study includes the oldest group of patients with dengue. These patients had high rates of comorbidities, including hypertension, diabetes mellitus, and chronic kidney disease. The in-hospital CFR was 41.3%, which is higher than in previous studies of patients with dengue in the ICUs [22, 23, 29, 30]. We found that cardiac arrest before ICU admission, AKI and prolonged APTT at ICU admission were independent prognostic factors for in-hospital fatality.

In the Philippines, DF is considered to be a pediatric disease [5] and severe dengue is recognized to be a leading cause of hospitalization and death among children in most Asian and Latin American countries [31]. The median age of indigenous cases with dengue over the past 25 years China is 39 years old [32], compared to 45 years in Taiwan from 2010 to 2012 [12]. In the current study, the median age of the patients was 72 years, which is higher than in other countries (Table 4). There is increasing awareness of DF affecting older adults in dengue endemic areas. Pang et al. summarized several recent clinical studies and indicated there was a shift of major affected age population of DENV, from children to adults [33], which was supported by a higher incidence in the adult population during the 2014–2015 outbreaks in Taiwan (S1 Fig).

**Table 4:** Mean or median age of cases of dengue fever in published articles.

1^st^ author, publication year [Reference]	Country	Study population	No. of cases	Age, year ^a^
**Lee, 2006** [17]	Taiwan	All	644	47.5±17.9
**Chandralekha, 2008** [29]	India	Intensive care unit	72	Range:13–65
**Juneja, 2011** [22]	India	Intensive care unit	198	39.6±17.1
**Pang, 2014** [30]	Singapore	Intensive care unit	27	46 [36–53]
**Kularatne, 2015** [9]	Sri Lanka	All	364	Mean: 30.2, range:16–63
**Le Viet, 2015** [10]	Vietnam	All	192	37 [24–48]
**Amancio, 2015** [23]	Brazil	Intensive care unit	97	42.6±20.3
**Hsieh [present study]**	Taiwan	Intensive care unit	75	72.3±9.3

^a^ Data are expressed as mean ± standard deviation or median [inter-quartile range], unless specified otherwise.

From the historical aspect of DF in Asia and Latin America, DF in the elderly in Taiwan is an emerging infectious disease and poses a new clinical challenge to physicians. Clinical data of the elderly with severe dengue requiring critical care are limited [22, 23]. A greater variation in the presentation of DF has been reported in elderly patients [14, 34], however the most pressing issue appears to be that aging is associated with severe dengue and DHF and thus poor outcomes [6, 35], consistent with the high mortality in the elderly patients with severe dengue in the current study. There are several possible reasons for this. First, the severity of disease in our cohort was high. Amancio *et al*. reported an in-hospital mortality rate of 19.6% in Brazilian adults admitted to an ICU with a median SOFA score of four and APACHE II score of eleven [23]. In contrast, the median APACHE II score in the present study was 20. The estimated CFR derived from the APACHE II score was 44.4% [26]. The standardized mortality ratio is 0.93 (41.4/44.4) in our study, and implies high standards of our ICU care.

Second, the cardiac and pulmonary reserve is usually limited in the elderly, as in our study cohort who had a mean age of 72 years. Intravenous fluid must be administered cautiously in the elderly to avoid cardiogenic pulmonary edema, however inadequate fluid replacement can worsen organ failure [9]. The physicians faced the dilemma of fluid therapy in such an elderly cohort. Third, the fact that seven cases experienced cardiac arrest before ICU admission may imply inadequate fluid resuscitation, which may be related to delayed access to medical care. Comprehensive public health education during dengue outbreaks should therefore be emphasized. Finally, Taiwanese tend to avoid invasive life-supporting interventions such as hemodialysis or endotracheal intubation for the elderly, and this limits their opportunity for recovery.

Several risk factors have been associated with ICU admission for patients with dengue. Pang et al. conducted a matched case-control study and found that diabetes mellitus, the WHO 2009 classification of severe dengue, hypoproteinemia, hematocrit change of at least 20% concurrent with fewer than 50,000/ml platelets, hypotension, and severe organ involvement were early clinical risk factors at presentation for ICU admission [30, 33]. In our cohort, 81.3% of the patients were classified as having severe dengue at presentation, and 42.7% of the patients had diabetes mellitus. The leading causes of ICU admission were gastrointestinal bleeding (30.7%), acute respiratory failure (24.0%) and shock (13.3%) in our cohort. These characteristics are compatible with the previous study [33].

More than 70% of our patients received mechanical ventilation, which had been associated with a poor prognosis in the literature [36, 37]. The respiratory and cardiovascular components of the SOFA score have been reported to be better predictors than other components of the SOFA score in one ROC analysis [23]. In accordance with a previous study [36], the causes of respiratory failure were diverse in our cohort, and some cases were intubated due to clinical concerns of volume overload during fluid resuscitation. In the Cox model, although respiratory failure indicative of a deterioration of clinical status and dysregulation in respiratory function, at ICU admission, was marginally significant, respiratory failure still remains to be an important prognostic factor. Other physiologic parameters, such as the ratio of arterial oxygen partial pressure to fractional inspired oxygen (PaO2/FiO2), *i*.*e*., the respiratory component of SOFA score predicted the fatality in a previous cohort study [22].

Old age, acute renal failure, acute respiratory failure, acute hepatitis, DSS/DHF, altered mental status, bleeding, and coagulopathy have been reported to be predictive factors for fatality in the patients with DF [19–21]. In addition, hypoalbuminemia and high serum lactate level have been associated with high mortality [38]. In our elderly cohort, AKI, cardiac arrest before ICU admission, and prolonged APTT were independent predictive factors for in-hospital fatality. However, we did not observe a similar role of other surrogate markers of coagulation including prothrombin time (PT) and platelet count. Previous studies have reported that APTT prolongation is a clinical predictor of DENV [39, 40] or DHF [41]. Of note, we found that APTT prolongation was a prognostic factor in our critically ill adults with severe dengue. DENV can cause derangement of the coagulation system [42], and APTT had more frequently been reported to be prolonged than PT in patients with DHF [43, 44]. Direct liver involvement by dengue virus is common in symptomatic patients with DF, and this can result in decreased production of coagulation factors [45]. The severity of DF has been associated with the extent of coagulation and fibrinolysis activation [43, 46]. In addition, a previous study demonstrated that dengue NS1 may bind to prothrombin, inhibit prothrombin activation and prolong APTT in vitro [47]. This may be a possible reason why APTT prolongation was an important factor in our cohort. However, the exact mechanisms underlying this APTT prolongation in severe dengue warrant further investigations.

The current 2009 WHO dengue case classification categorizes dengue patients into three groups: dengue without warning signs, dengue with warning signs, and severe dengue [24]. This classification has improved the triage and management of dengue, compared to the 1997 WHO dengue classification [48, 49]. However, there is still room for improvement from a clinical perspective, especially with regards to risk stratification. The warning signs are too broad and include too many patients at risk [49]. From the results of the current study, APTT may be considered as a screening tool for risk stratification. The incorporation of more subjective laboratory tests into the classification framework may facilitate effective patient triage in dengue endemics.

### Limitations

There are several limitations to this study. First, this is a retrospective study conducted in the ICUs of a single tertiary hospital, in which a high severity of disease and high selection bias could be expected. The conclusions cannot be generalized to all age groups, especially pediatric populations. Second, the number of cases is too limited to include many variables in one analysis. Accordingly, the confidence intervals of the hazard ratios in the Cox model are wide. Third, only the laboratory data on the first day of ICU admission were included for analysis, and their dynamic changes, which may signify additional clinical impact, were not considered. However, we believe that the early prognostic significance resulting from initial laboratory abnormalities at the time of ICU admission will be valuable for intensive care physicians when treating elderly patients with severe dengue. Finally, though APTT prolongation can be an early marker of in-hospital fatality, it remains obscure that more or early intensive care can improve the outcome of dengue patients with APTT prolongation. Prospective clinical studies are warranted to verify the benefit of early risk stratification of the elderly with severe dengue.

## Conclusion

During the 2015 dengue outbreak in Taiwan, the patients with severe dengue in ICUs were characterized by old age, multiple comorbidities, and a high CFR. Organ failure (including cardiac failure and renal failure) and coagulation disturbance (prolongation of initial APTT) were independent predictive factors for in-hospital fatality. Further studies are needed to clarify the relationship and mechanism between APTT prolongation and in-hospital fatality in patients with DF.

## Supporting Information

S1 Checklist STROBE Checklist. (DOCX)

S1 Dataset The dataset of this study. (XLS)

S2 Dataset The dataset of the fatality cases. (XLS)

S1 Fig The age distribution of nationwide dengue cases over 2014 and 2015 in Taiwan. (PDF)

S2 Fig The Kaplan-Meier curves of each dichotomous predictive factor. (TIF)

S1 File The Cox models and ROC analysis. (PDF)
