# Supplementary material for: A Cohort Study of Adult Patients with Severe Dengue in Taiwanese Intensive Care Units: The Elderly and APTT Prolongation Matter for Prognosis
Source: PLoS Negl Trop Dis. 2017 Jan 6;11(1):e0005270. doi: 10.1371/journal.pntd.0005270 (PMC5245902; doi:10.1371/journal.pntd.0005270)
Supplement: S1 Fig — (PDF) [file pntd.0005270.s004.pdf]

The age distribution of dengue outbreak over 2014 and 2015 in Taiwan [1].

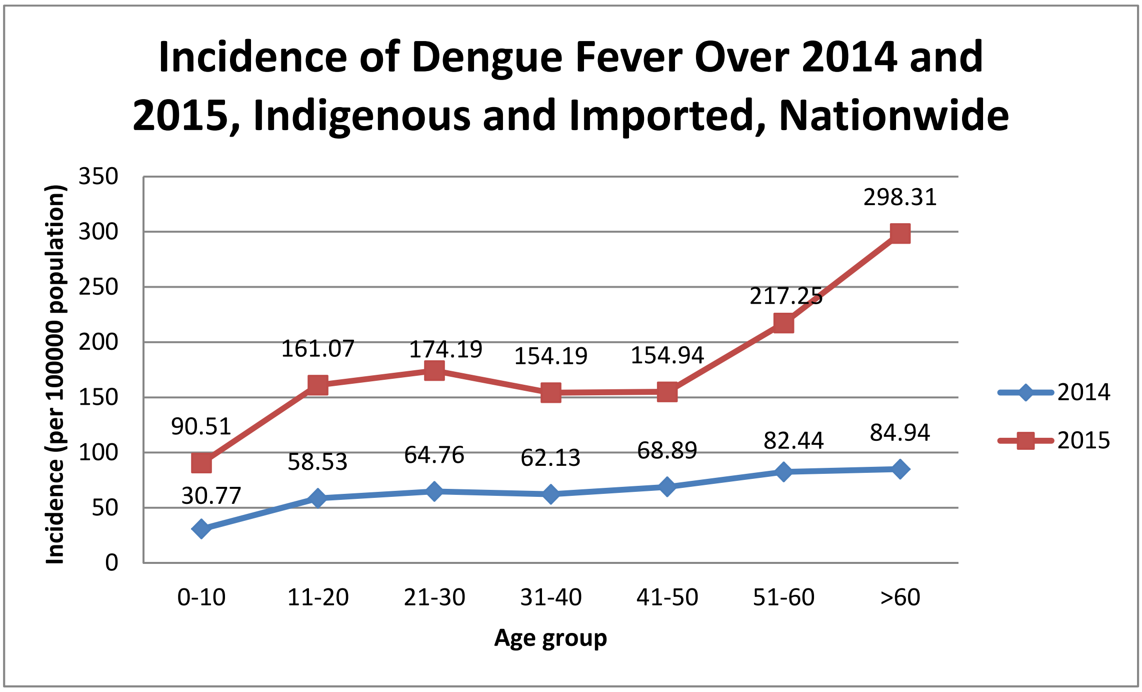

1. Center for Disease Control (Taiwan) : Taiwan National Infectious Disease Statistics System for Dengue Virus Surveillance. Taiwan: CDC, 2015. Available at http://nidss.cdc.gov.tw/en/NIDSS_Diagram.aspx?dc=1&dt=4&disease=061 (accessed 14 Nov 2016)
