# Supplementary figures and images for: A Cohort Study of Adult Patients with Severe Dengue in Taiwanese Intensive Care Units: The Elderly and APTT Prolongation Matter for Prognosis
Source: PLoS Negl Trop Dis. 2017 Jan 6;11(1):e0005270. doi: 10.1371/journal.pntd.0005270 (PMC5245902; doi:10.1371/journal.pntd.0005270)

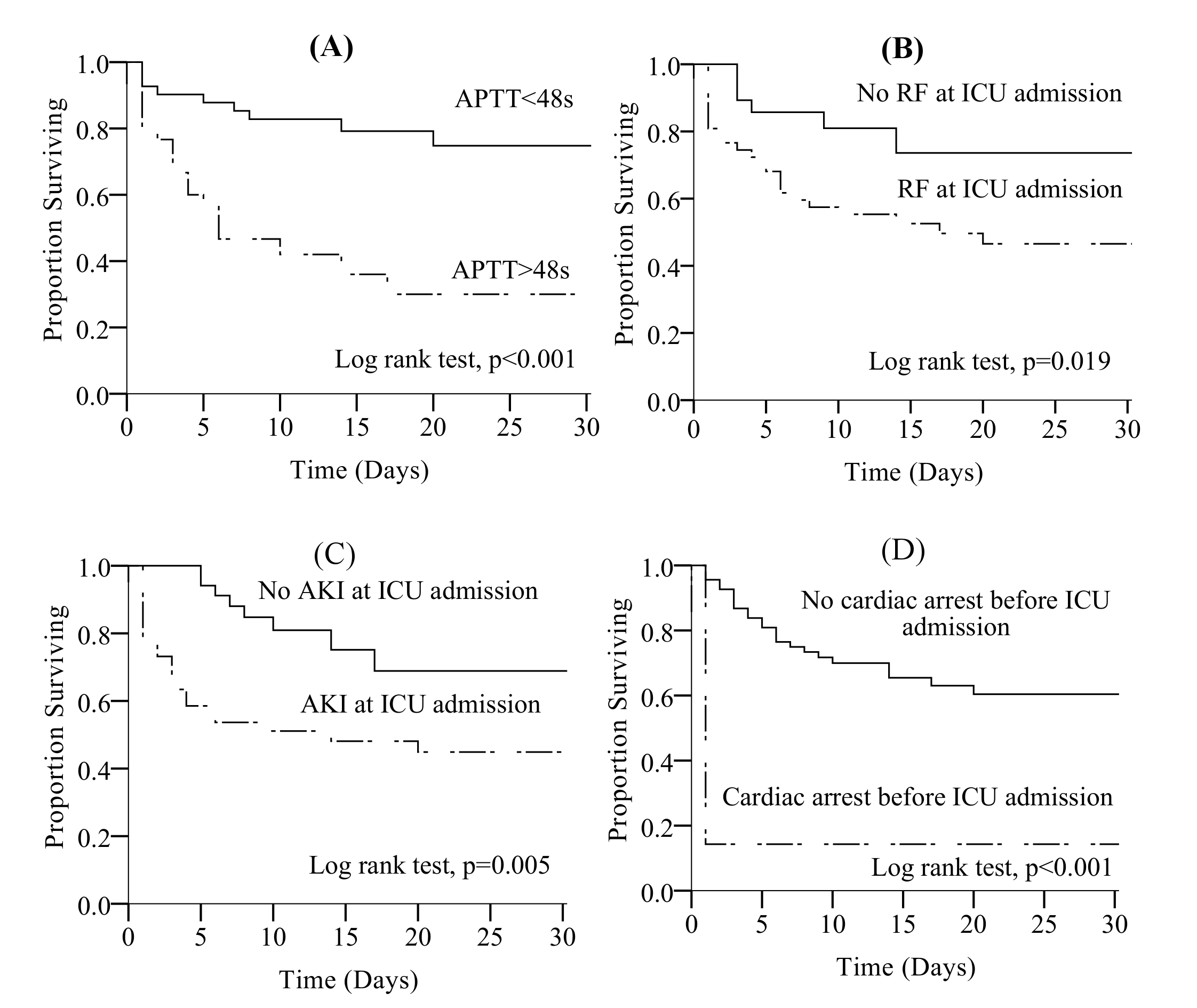

Supplement: S2 Fig — (TIF) [file pntd.0005270.s005.tif]
