# Supplementary material for: A Cohort Study of Adult Patients with Severe Dengue in Taiwanese Intensive Care Units: The Elderly and APTT Prolongation Matter for Prognosis
Source: PLoS Negl Trop Dis. 2017 Jan 6;11(1):e0005270. doi: 10.1371/journal.pntd.0005270 (PMC5245902; doi:10.1371/journal.pntd.0005270)
Supplement: S1 File — (PDF) [file pntd.0005270.s006.pdf]

The formation of the Cox model

Due to a limited case number, we set up the Cox model with a limited number of the variables to meet the rule of "one in ten". Spearman's correlation matrix was constructed for all significant variables in the univariate Cox model. To avoid collinearity, only independent variables were selected. Serum creatinine and acute renal failure were highly correlated, and SOFA score and APACHE II score correlated with most variables. Thus, we did not include serum creatinine, SOFA and APACHE II scores in the final model. However, some analyses were performed to observe the changes of hazard ratio of APTT prolongation.

1. Supplementary Table 2A showed an over-adjusted model 1, which included both SOFA and APACHE II scores. As a result, the only significant variable was SOFA score.
2. Supplement Table 2B showed the model 2, which included APACHE II score. APACHE II score, cardiac arrest, and APTT prolongation were the significant variables.
3. Supplement Table 2C showed the model 3, which included SOFA score. Only gender and SOFA score achieved significance, and APTT prolongation was marginally significant (*P*= 0.053).
4. Supplement Table 2D showed the model 4, which included serum creatinine and acute kidney injury at ICU admission, and was contained the least patient number because of missing data of serum creatinine. In contrast, acute kidney injury at ICU admission was a better variable than serum creatinine for inclusion in the final model.
5. Supplement Table 2E showed the model 5, which contained gender, acute kidney injury and respiratory failure at ICU admission, cardiac arrest before ICU admission, and APTT prolongation. We chose this model as the final model because these variables were mutually independent in terms of the statistics and clinical consideration.

Supplementary Table 1: Univariate Cox regression.

Variables	Case number	HR (unadjusted)	*P* value
Age > 70 years	75	1.71 [0.74-3.98]	0.211
Age (per year)	75	1.05 [1.00-1.09]	0.05
Male gender	75	0.41 [0.20-0.84]	0.015
Body weight (per kg)	75	0.97 [0.95-1.00]	0.066
Diabetes mellitus	75	1.02 [0.50-2.09]	0.949
Hypertension	75	0.57 [0.27-1.17]	0.123
Chronic kidney disease	75	1.62 [0.74-3.52]	0.224
Chronic obstructive lung disease	75	0.25 [0.03-1.80]	0.168
Coronary artery disease	75	1.13 [0.46-2.76]	0.768
Congestive heart failure	75	1.20 [0.36-3.96]	0.768
Malignancy	75	1.70 [0.70-4.18]	0.245
Dyslipidemia	75	1.23 [0.53-2.85]	0.634
Stroke	75	0.56 [0.17-1.87]	0.349
Chronic liver disease	75	0.69 [0.16-2.93]	0.617
APACHE II score (per point)	75	1.09 [1.06-1.12]	<0.001
APACHEII score > 24	75	4.70 [2.20-10.03]	<0.001
SOFA score (per point)	75	1.26 [1.15-1.38]	<0.001
SOFA score > 15	75	7.56 [3.65-15.64]	<0.001
AKI at ICU admission	75	2.95 [1.32-6.63]	0.009
RF at ICU admission	75	2.73 [1.12-6.67]	0.027
CA before ICU admission	75	8.08 [3.06-21.33]	<0.001
APTT > 48 seconds	71	4.54 [2.04-10.07]	<0.001
APTT (per second)	71	1.07 [1.04-1.11]	<0.001
PT (per seconds)	72	1.03 [0.98-1.08]	0.227
Hemoglobin (g/dL)	75	0.93 [0.83-1.06]	0.273
Hematocrit (%)	75	0.98 [0.94-1.02]	0.301
Platelet (x1000/dL)	75	1.00 [0.99-1.00]	0.214
Total bilirubin (mg/dL)	60	1.07 [0.84-1.35]	0.604
Creatinine (mg/dL)	72	1.18 [1.03-1.34]	0.016
Creatinine > 1.5 mg/dL	72	2.52 [1.18-5.39]	0.017
AST (U/L)	71	1 [1-1]	0.275
ALT (U/L)	74	1 [1-1]	0.751

Supplementary Table 2A: Multivariate Cox regression model 1 included gender, APACHE II and SOFA sore, acute kidney failure (AKI), respiratory failure, cardiac arrest, and APTT prolongation (n =71).

Variables	HR (unadjusted)	*P* value	HR (adjusted)	*P* value
Gender (male)	0.41 [0.20-0.84]	0.015	0.43 [0.18-1.01]	0.054
APACHE II score > 24	4.70 [2.20-10.03]	<0.001	2.03 [0.60-6.86]	0.254
SOFA score > 15	7.56 [3.65-15.64]	<0.001	4.71 [0.60-6.86]	0.022
AKI at ICU admission	2.95 [1.32-6.63]	0.009	0.86 [0.26-2.80]	0.765
RF at ICU admission	2.73 [1.12-6.67]	0.027	1.48 [0.45-4.82]	0.515
CA before ICU admission	8.08 [3.06-21.33]	<0.001	3.28 [0.92-11.7]	0.067
APTT > 48 seconds	4.54 [2.04-10.07]	<0.001	2.35 [0.99-5.58]	0.052

Supplementary Table 2B: Multivariate Cox regression model 2 included gender, APACHE II score, acute kidney failure (AKI), respiratory failure, cardiac arrest, and APTT prolongation (n=71).

Variables	HR (unadjusted)	*P* value	HR (adjusted)	*P* value
Gender (male)	0.41 [0.20-0.84]	0.015	0.61 [0.27-1.39]	0.236
APACHE II score > 24	4.70 [2.20-10.03]	<0.001	3.34 [1.25-8.91]	0.016
AKI at ICU admission	2.95 [1.32-6.63]	0.009	1.42 [0.53-3.80]	0.49
RF at ICU admission	2.73 [1.12-6.67]	0.027	1.84 [0.59-5.76]	0.295
CA before ICU admission	8.08 [3.06-21.33]	<0.001	5.34 [1.56-18.30]	0.008
APTT > 48 seconds	4.54 [2.04-10.07]	<0.001	3.34 [1.42-7.84]	0.006

Supplementary Table 2C: Multivariate Cox regression model 3 included gender, SOFA score, respiratory failure, cardiac arrest, and APTT prolongation (n=71).

Variables	HR (unadjusted)	*P* value	HR (adjusted)	*P* value
Gender (male)	0.41 [0.20-0.84]	0.015	0.42 [0.18-0.96]	0.041
SOFA score > 15	7.56 [3.65-15.64]	<0.001	6.93 [2.07-23.18]	0.002
AKI at ICU admission	2.95 [1.32-6.63]	0.009	1.01 [0.33-3.13]	0.987
RF at ICU admission	2.73 [1.12-6.67]	0.027	1.69 [0.53-5.35]	0.372
CA before ICU admission	8.08 [3.06-21.33]	<0.001	3.14 [0.90-10.96]	0.073
APTT > 48 seconds	4.54 [2.04-10.07]	<0.001	2.34 [0.99-5.53]	0.053

Supplementary Table 2D: Multivariate Cox regression model 4 included gender, acute kidney failure (AKI), serum creatinine, respiratory failure, cardiac arrest, and APTT prolongation (n=68).

Variables	HR (unadjusted)	*P* value	HR (adjusted)	*P* value
Gender (male)	0.41 [0.20-0.84]	0.015	0.69 [0.30-1.60]	0.389
AKI at ICU admission	2.95 [1.32-6.63]	0.009	4.17 [0.93-18.75]	0.063
Serum creatinine > 1.5 mg/dL	2.52 [1.18-5.39]	0.017	0.62 [0.15-25.3]	0.500
RF at ICU admission	2.73 [1.12-6.67]	0.027	3.35 [0.98-11.44]	0.053
CA before ICU admission	8.08 [3.06-21.33]	<0.001	6.70 [1.96-22.95]	0.002
APTT > 48 seconds	4.5 4 [2.04-10.07]	<0.001	4.75 [1.86-12.11]	0.001

Supplementary Table 2E: Multivariate Cox regression model 5 included gender, acute kidney failure (AKI), respiratory failure, cardiac arrest, and APTT prolongation (n=71).

Variables	HR (unadjusted)	*P* value	HR (adjusted)	*P* value
Gender (male)	0.41 [0.20-0.84]	0.015	0.66 [0.30-1.45]	0.298
AKI at ICU admission	2.95 [1.32-6.63]	0.009	2.48 [1.07-5.74]	0.035
RF at ICU admission	2.73 [1.12-6.67]	0.027	2.69 [0.91-7.95]	0.073
CA before ICU admission	8.08 [3.06-21.33]	<0.001	6.26 [1.91-20.54]	0.003
APTT > 48 seconds	4.54 [2.04-10.07]	<0.001	3.91 [1.69-9.07]	0.001

Supplementary Table 3: Spearman's correlation matrix of potential variables in the Cox regression model.

Variables	APACHE II score	SOFA score	Serum creatinine	Male gender	AKI	RF	CA	APTT > 48 s
APACHE II score	1	0.644*	0.481*	-0.100	0.448*	0.330*	0.310*	0.152*
SOFA score	-	1	0.449*	-0.003	0.449*	0.369*	0.464*	0.322*
Serum creatinine	-	-	1	-0.057	0.757*	0.000	0.234*	0.145
Gender	-	-	-	1	0.047	-0.047	-0.216	-0.243*
AKI	-	-	-	-	1	0.128	0.200	0.144
RF	-	-	-	-	-	1	0.180	0.012
CA	-	-	-	-	-	-	1	0.100
APTT > 48 s	-	-	-	-	--	--	--	1

**P* value < 0.05.

Supplementary Table 4: Receiver operating characteristic curve analysis**.**

Variables	AUC	95% CI	*P* value
SOFA score (per point)	0.85	0.77-0.94	<0.001
APACHE II score (per point)	0.84	0.75-0.93	<0.001
APACHE II score > 24	0.76	0.64-0.88	<0.001
SOFA score > 15	0.76	0.64-0.89	<0.001
APTT (per second)	0.76	0.64-0.88	<0.001
APTT > 48 seconds	0.73	0.60-0.85	0.001
RF at ICU admission	0.68	0.56-0.81	0.010
AKI at ICU admission	0.65	0.52-0.78	0.035
Creatinine (mg/dL)	0.64	0.50-0.78	0.053
Creatinine > 1.5mg/dL	0.63	0.50-0.77	0.062
Age (per year)	0.60	0.42-0.69	0.069
CA before ICU admission	0.59	0.45-0.73	0.192
Age > 70 years	0.56	0.42-0.69	0.069
Gender (male)	0.37	0.23-0.50	0.068

Abbreviations:

AKI = acute kidney injury; ALT = alanine transaminase; APACHE II = acute physiology and chronic health evaluation II; APTT = activated partial thromboplastin time; RF= respiratory failure; AST = aspartate transaminase; AUC = area under curve; CA = cardiac arrest; CI = confidence interval; HR = hazard ratio; ICU= intensive care unit; PT = prothrombin time; SOFA = sequential organ failure assessment.
